# Endothelial Progenitor Cells: Relevant Players in the Vasculopathy and Lung Fibrosis Associated with the Presence of Interstitial Lung Disease in Systemic Sclerosis Patients

**DOI:** 10.3390/biomedicines9070847

**Published:** 2021-07-20

**Authors:** Verónica Pulito-Cueto, Sara Remuzgo-Martínez, Fernanda Genre, Belén Atienza-Mateo, Víctor M. Mora-Cuesta, David Iturbe-Fernández, Leticia Lera-Gómez, Raquel Pérez-Fernández, Diana Prieto-Peña, Virginia Portilla, Ricardo Blanco, Alfonso Corrales, Oreste Gualillo, José M. Cifrián, Raquel López-Mejías, Miguel A. González-Gay

**Affiliations:** 1 Research Group on Genetic Epidemiology and Atherosclerosis in Systemic Diseases and in Metabolic Bone Diseases of the Musculoskeletal System, IDIVAL, 39011 Santander, Spain; veronica_pulito_cueto@hotmail.com (V.P.-C.); sararmtz@gmail.com (S.R.-M.); fernandagenre@gmail.com (F.G.); mateoatienzabelen@gmail.com (B.A.-M.); victormanuel.mora@scsalud.es (V.M.M.-C.); diturfer@gmail.com (D.I.-F.); letizialera@hotmail.com (L.L.-G.); kelyra95@hotmail.com (R.P.-F.); diana.prieto.pena@gmail.com (D.P.-P.); virgiportilla@hotmail.com (V.P.); ricardo.blanco@scsalud.es (R.B.); afcorralesm@hotmail.com (A.C.); josemanuel.cifrian@scsalud.es (J.M.C.); 2 López Albo’ Post-Residency Programme, Hospital Universitario Marqués de Valdecilla, 39008 Santander, Spain; 3 Department of Rheumatology, Hospital Universitario Marqués de Valdecilla, 39008 Santander, Spain; 4 Department of Pneumology, Hospital Universitario Marqués de Valdecilla, 39008 Santander, Spain; 5 Servizo Galego de Saude and Instituto de Investigación Sanitaria, Hospital Clinico Universitario de Santiago, 15706 Santiago de Compostela, Spain; orestegualillo@gmail.com; 6 School of Medicine, Universidad de Cantabria, 39011 Santander, Spain; 7 Cardiovascular Pathophysiology and Genomics Research Unit, School of Physiology, Faculty of Health Sciences, University of the Witwatersrand, Johannesburg 2050, South Africa

**Keywords:** endothelial progenitor cells, systemic sclerosis, interstitial lung disease, biomarker

## Abstract

Endothelial progenitor cells (EPC), which are key effectors in the physiologic vascular network, have been described as relevant players in autoimmune diseases. We previously showed that EPC frequency may help to identify the presence of interstitial lung disease (ILD) in rheumatoid arthritis patients. Given that ILD constitutes the main cause of mortality in systemic sclerosis (SSc) patients, we aimed to determine the EPC contribution to the pathogenic processes of vasculopathy and lung fibrosis in SSc-ILD^+^. EPC quantification was performed by flow cytometry on blood from 83 individuals: 21 SSc-ILD^+^ patients and subjects from comparative groups (20 SSc-ILD^−^ and 21 idiopathic pulmonary fibrosis (IPF) patients and 21 healthy controls (HC)). EPC were considered as CD34^+^, CD45^low^, CD309^+^, and CD133^+^. A significant increase in EPC frequency was found in SSc-ILD^+^ patients when compared to HC (*p* < 0.001). SSc-ILD^+^ patients exhibited a higher EPC frequency than SSc-ILD^−^ patients (*p* = 0.012), whereas it was markedly reduced compared to IPF patients (*p* < 0.001). EPC frequency was higher in males (*p* = 0.04) and negatively correlated to SSc duration (*p* = 0.04) in SSc-ILD^+^ patients. Our results indicate a role of EPC in the processes of vasculopathy and lung fibrosis in SSc-ILD^+^. EPC frequency may be considered as a biomarker of ILD in SSc patients.

## 1. Introduction

Endothelial progenitor cells (EPC) are known to be key cellular effectors in the homeostasis of the physiologic vascular network, being implicated in vascular regeneration, both in new vessel formation and in the repair mechanisms of existing vessels [1,2,3]. The ability of these cells to differentiate towards mature endothelial cells and to incorporate into the injured vasculature, promoting neovascularization, has raised great interest over the last several decades [2,4]. Accordingly, growing evidence has shown the relevant contribution of EPC to the pathogenesis of different vascular diseases [2,4,5,6]. In fact, we recently proposed that the degree of EPC frequency may be useful as a biomarker to identify the presence of interstitial lung disease (ILD) in patients with rheumatoid arthritis (RA) [7].

Systemic sclerosis (SSc) is a clinically heterogeneous disease characterized by a complex interplay between autoimmunity, vasculopathy, and fibrosis [8,9,10,11,12]. ILD is one of the most severe and common manifestations of SSc and a leading cause of death in these patients [8,9,10,11,12]. However, the options for treatment are limited, since the underlying mechanisms of defective vasculogenesis and lung fibrosis of SSc-ILD are not clear [12]. Therefore, the investigation of novel, reliable, and non-invasive biomarkers would provide a better understanding of the pathophysiology of SSc-ILD [10,12]. In this context, EPC have been described as important players in the pathogenesis of SSc [13,14,15,16,17,18,19,20,21,22,23,24,25,26,27,28,29,30]. Nevertheless, to the best of our knowledge, their role in the development of ILD in SSc patients remains unknown.

Taking all this into account and given the essential role of EPC in endothelial repair, the objective of this study was to determine the contribution of EPC to the pathogenic processes of vasculopathy and lung fibrosis in SSc-ILD^+^.

## 2. Materials and Methods

### 2.1. Study Population

A total of 83 individuals constituted by 21 SSc-ILD^+^ patients and subjects from three comparative groups (20 SSc-ILD^−^ patients, 21 idiopathic pulmonary fibrosis (IPF) patients, and 21 healthy controls (HC)) were recruited from the Pneumology and Rheumatology departments of Hospital Universitario Marqués de Valdecilla (Santander, Spain).

Patients with SSc fulfilled the 2013 American College of Rheumatology/European League Against Rheumatism criteria for the classification of SSc [31]. Pulmonary fibrosis was assessed in all the patients by high-resolution computed tomography (HRCT) images of the chest and pulmonary function tests (PFTs). Additionally, pulmonary hypertension (PH) was diagnosed by transthoracic echocardiogram in all the patients. SSc patients who lacked lung involvement (absence of pulmonary fibrosis and PH) were considered as SSc-ILD^−^ patients, whereas those who fulfilled the American Thoracic Society/European Respiratory Society criteria for ILD were classified as SSc-ILD^+^ [32]. IPF patients met the criteria proposed by the American Thoracic Society/European Respiratory Society [32]. HC did not present any history of autoimmune or lung diseases.

For further clinical characterization, demographic and clinical features of patients including sex, age, smoking history, duration of SSc disease (early: ≤ 5 years; late: >5 years), antibodies status, C-reactive protein, erythrocyte sedimentation rate, PFTs, PH, pulmonary fibrosis on HRCT, HRCT patterns, and other SSc clinical manifestations at the time of the study were collected (Table 1). HRCT patterns of ILD patients were stratified according to the Fleischner society’s criteria for usual interstitial pneumonia pattern [33].

All the experiments involving humans and human blood samples were carried out in accordance with the approved guidelines and regulations, according to the Declaration of Helsinki. All experimental protocols were approved by the Ethics Committee of Clinical Research of Cantabria, Spain (2016.092). All subjects gave written informed consent to participate in this study prior to their inclusion.

### 2.2. EPC Quantification by Flow Cytometry

EPC from peripheral venous blood were characterized by simultaneous expression of cell surface markers that reflect stemness (CD34), immaturity (CD133), endothelial commitment (CD309 or vascular endothelial growth factor receptor 2 (VEGFR-2)), and a low expression of the pan-leukocyte marker (CD45) [34,35,36].

EPC quantification was analyzed by direct flow cytometry following recommendations on EPC measurement from the EULAR Scleroderma Trials and Research (EUSTAR) group and other methods previously described [34,35,36]. Briefly, 200 µL of peripheral blood was pre-incubated with FcR blocking reagent (Miltenyi Biotech, Madrid, Spain). Then, cells were labeled with APC-conjugated anti-CD34 (Miltenyi Biotech, Madrid, Spain), VioBright FITC-conjugated anti-CD309 (VEGFR-2) (Miltenyi Biotech, Madrid, Spain), PE-conjugated anti-CD133/2(293C3) (Miltenyi Biotech, Madrid, Spain), and VioBlue-conjugated anti-CD45 (Miltenyi Biotech, Madrid, Spain) monoclonal antibodies. Specificity of staining was controlled by incubation with isotype-matched antibodies (Miltenyi Biotech, Madrid, Spain). After conjugation, red blood cells were lysed by incubating in FACS lysing solution (BD Bioscience, San Jose, CA, USA) and white blood cell pellets were then washed once with PBS. Labeled cells were analyzed in a CytoFLEX flow cytometer (Beckman Coulter, Brea, CA, USA) using a Cytexpert 2.3 analyzer (Beckman Coulter, Brea, CA, USA), acquiring approximately 1 × 10^5^ eventsper sample. First, CD34^+^ and CD45^low^ were gated and then assayed for expression of CD133 and CD309 in the lymphocyte gate. Thus, EPC were considered as CD34^+^, CD45^low^, CD133^+^ and CD309^+^ cells. EPC quantification was expressed as the percentage of cells in the lymphocyte gate.

### 2.3. Statistical Analyses

Data were expressed as mean ± standard deviation (SD) for continuous variables and as number of individuals (*n*) and percentage (%) for categorical variables. Comparisons of EPC frequency between two study groups were performed by Student’s *t*-test. The relationships of EPC frequency with continuous variables and categorical variables related to demographic and disease features were established via estimation of Pearson’s correlation coefficient (r) and one-way ANOVA, respectively. *p*-values < 0.05 were considered as statistically significant. Statistical analysis was performed using STATA 12/SE statistical software (Stata Corp., College Station, TX, USA).

## 3. Results

### 3.1. Differences in EPC Frequency between SSc-ILD^+^ Patients and the Comparative Groups

**Figure 1:**
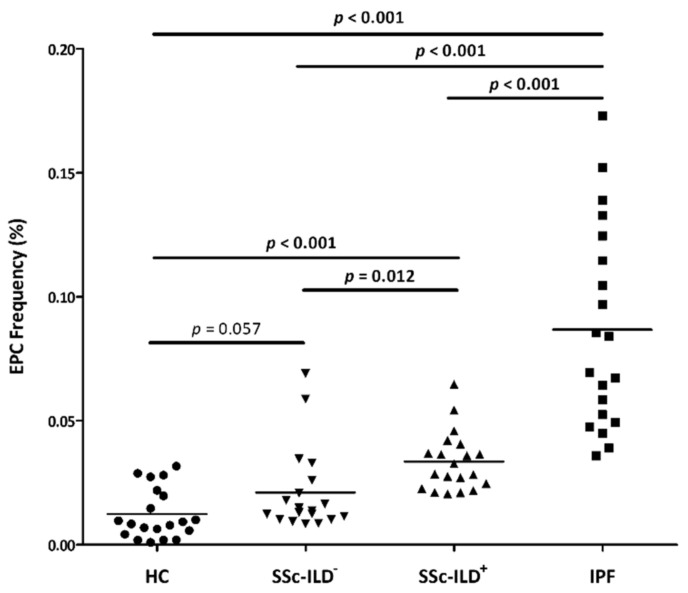
Quantification of EPC population by flow cytometry in all individuals included in the study. EPC were considered as CD34^+^, CD45^low^, CD309^+^, and CD133^+^ cells in the lymphocyte gate and are expressed as the percentage of cells in this gate. Differences between the study groups were evaluated by Student’s *t*-test. *p*-values <0.05 were considered as statistically significant. Horizontal bars indicate the mean value of each study group. EPC: endothelial progenitor cells; HC: healthy controls; SSc: systemic sclerosis; ILD: interstitial lung disease; IPF: idiopathic pulmonary fibrosis.

Patients with SSc-ILD^+^ showed a significantly higher EPC frequency than HC (*p* < 0.001) (Figure 1 and Table S1). Likewise, EPC frequencies were increased in IPF and SSc-ILD^−^ patients when compared to HC, but in the latter case it was marginally statistically significant (*p* < 0.001 and *p* = 0.057, respectively) (Figure 1).

Moreover, EPC frequency was increased in SSc-ILD^+^ patients compared to patients with SSc-ILD^−^ (*p* = 0.012), whereas it was markedly reduced compared to IPF patients (*p* < 0.001) (Figure 1 and Table S1). Furthermore, patients with SSc-ILD^−^ exhibited a lower frequency of EPC than IPF patients (*p* < 0.001) (Figure 1).

**Figure 2:**
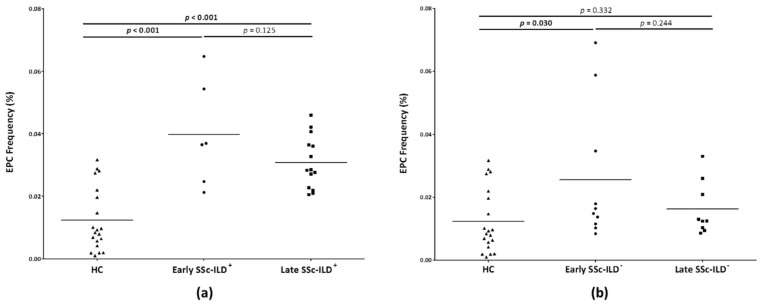
Quantification of EPC population by flow cytometry in HC and patients with SSc-ILD^+^ and SSc-ILD^−^ stratified according to SSc duration. (**a**) EPC frequency in HC and patients with early and late SSc-ILD^+^. (**b**) EPC frequency in HC and patients with early and late SSc-ILD^−^. EPC were considered as CD34^+^, CD45^low^, CD309^+^, and CD133^+^ cells in the lymphocyte gate and are expressed as the percentage of cells in this gate. Differences between the study groups were evaluated by Student’s *t*-test. *p*-values < 0.05 were considered as statistically significant. Horizontal bars indicate the mean value of each study group. EPC: endothelial progenitor cells; HC: healthy controls; SSc: systemic sclerosis; ILD: interstitial lung disease.

In a further step, SSc-ILD^+^ and SSc-ILD^−^ patients were stratified by disease duration. In this sense, EPC frequency was greater both in early and late SSc-ILD^+^ patients when compared to HC (*p* < 0.001 in both cases) (Figure 2a). No significant differences were observed between early and late SSc-ILD^+^ patients (Figure 2a). Regarding SSc-ILD^−^, early SSc-ILD^−^ patients showed EPC frequencies significantly greater than HC (*p* = 0.030), while no differences were found between late SSc-ILD^−^ patients and HC (*p* = 0.332) (Figure 2b). No significant differences were observed between early and late SSc-ILD^−^ patients (Figure 2b).

### 3.2. Relationship of EPC Frequency with Demographic and Clinical Features

We found a negative correlation between the frequency of EPC and the SSc disease duration in patients with SSc-ILD^+^ (r = −0.45; *p* = 0.04) (Table 2). Moreover, EPC frequency was higher in male SSc-ILD^+^ patients when compared to female patients (*p* = 0.04) (Table 3). No significant relationship between EPC frequency and the other demographic and clinical features assessed was found in SSc-ILD^+^ patients (Table 2 and Table 3).

Regarding patients with SSc-ILD^−^ and IPF, no significant results were obtained (Table 2 and Table 3).

## 4. Discussion

EPC have been described as important players in the pathogenesis of vascular diseases [2,4,5,6]. Given that we recently identified EPC as a potential biomarker of endothelial damage in RA-ILD^+^ [7], and based on the relevance of ILD as a main cause of mortality in patients with SSc [8,9,10,11,12], we wondered if EPC may also play a crucial role in SSc-ILD^+^. Accordingly, we assessed the contribution of EPC to the pathogenic processes of vasculopathy and lung fibrosis in SSc-ILD^+^.

Our study found a relevant role of EPC in the vasculopathy process of SSc-ILD^+^. In particular, we found an increase in EPC production and mobilization into the systemic circulation in SSc-ILD^+^ patients, since a higher EPC frequency was found in these patients when compared to HC. This difference remained significant when SSc-ILD^+^ patients were stratified into early and late disease duration. Given that endothelial injury and insufficient endothelial repair are strong contributors to the vasculopathy underlying SSc-ILD^+^, we hypothesize that EPC are increasing in peripheral blood to be recruited at the sites of vascular damage and to exert their reparative function as a compensatory mechanism. This finding is consistent with a previous result of our group from a study performed in RA-ILD^+^ patients [7]. Likewise, other groups demonstrated an increase in circulating EPC in SSc patients in relation to HC [14,15,16,20,26,27,28,30], mainly in the early stage of the disease [14,20,26,27,28]. In keeping with this, our study showed significantly greater EPC frequencies in early SSc-ILD^−^ patients than HC, unlike late SSc-ILD^−^ patients. The latter may explain the lack of statistically significant difference between HC and SSc-ILD^−^ patients, regardless of SSc duration. We also observed an increase in EPC frequency in IPF patients in relation to HC, as described in a previous work [6], further supporting the compensatory mechanism of these cells.

Interestingly, an increase in EPC linked to the presence and severity of ILD was found in our study. According to this, patients with SSc-ILD^+^ exhibited a higher EPC frequency than those patients with SSc-ILD^−^ who did not present lung disease. Notably, the greatest EPC frequencies were found in IPF patients, who experience the most aggressive form of ILD. This behavior of EPC is similar to that observed in our previous work, in which we showed that the degree of EPC frequency may help to identify the presence of ILD in RA patients [7]. Therefore, although the exact mechanism responsible for these findings is not clear, it can be speculated that it may be associated with an enhancement of EPC production in response to the lung fibrotic process and, consequently, to the presence of ILD in patients with autoimmune diseases. In favor of this suggestion, previous reports in SSc indicated an association of EPC with lung involvement [15,16]. Our results further support the idea that EPC frequency may be considered as a useful complementary tool to identify the presence of ILD in SSc patients, mirroring the severity of SSc disease.

It is known that several factors influence the development of SSc-ILD^+^, including shorter disease duration and male sex [9,11]. Notably, our results revealed an inverse correlation of EPC frequency with SSc disease duration in SSc-ILD^+^ patients, as reported in SSc by different authors [16,20,25,34]. It is conceivable that the decrease in EPC in patients with long-standing disease is related to the recruitment of such cells into damaged tissues, leading to a decrease in circulating EPC. Furthermore, we found a higher frequency of EPC in men, which seems to be expected considering that the male sex is a known SSc-ILD^+^ risk factor. Nevertheless, no further association was found between EPC and demographic or disease features in SSc-ILD^+^ patients or in SSc-ILD^−^ and IPF patients. In agreement with this, previous reports showed a lack of association of EPC with PFTs and smoking status in IPF patients [37], as well as with age, inflammation markers, antibodies status, and PH presence in SSc patients [14,19,24,25,27].

In conclusion, our findings provide evidence for a potential role of EPC in the pathogenic processes of vasculopathy and lung fibrosis in SSc-ILD^+^. Interestingly, EPC frequency may be considered a promising marker for vascular damage and disease progression, particularly regarding the presence of ILD in patients with SSc.

## Figures and Tables

**Table 1:** Demographic and clinical features of the individuals included in this study.

	SSc-ILD^+^ Patients *n* = 21	SSc-ILD^−^ Patients *n* = 20	IPF Patients *n* = 21	Healthy Controls *n* = 21
Sex (women), *n* (%)	13 (61.9)	18 (90.0)	7 (33.3)	7 (33.3)
Age at study, mean ± SD, years	60.3 ± 7.0	56.6 ± 15.4	69.2 ± 10.0	41.2 ± 12.5
Smoking ever, *n* (%)	11 (52.4)	11 (55.0)	16 (76.2)	5 (31.3)
SSc duration, mean ± SD, years	10.8 ± 8.3	9.6 ± 8.1	-	-
**Antibodies status**				
ANA positive, *n* (%)	19 (95.0)	18 (90.0)	-	-
ACA positive, *n* (%)	1 (5.0)	9 (45.0)	-	-
ATA (anti-Scl70) positive (%)	10 (50.0)	4 (20.0)	-	-
CRP (mg/dL), mean ± SD	0.7 ± 1.4	0.5 ± 0.5	-	-
ESR (mm/1st hour), mean ± SD	20.1 ± 15.9	17.2 ± 13.4	-	-
**Pulmonary function tests**				
FVC (% predicted), mean ± SD	88.4 ± 27.1	106.6 ± 15.9	84.9 ± 14.7	-
FEV1 (% predicted), mean ± SD	87.3 ± 25.6	101.9 ± 17.8	87.3 ± 19.6	-
FEV1/FVC (% predicted), mean ± SD	79.7 ± 5.5	79.2 ± 9.9	79.7 ± 7.8	-
DLCO (% predicted), mean ± SD	47.5 ± 19.5	71.5 ± 15.3	43.6 ± 18.4	-
Pulmonary hypertension, *n* (%)	3 (15.8)	0 (0.0)	4 (26.7)	-
**HRCT**				
Pulmonary involvement on HRCT	21 (100.0)	0 (0.0)	21 (100.0)	-
NSIP pattern, *n* (%)	14 (66.7)	-	0 (0.0)	-
Non-NSIP pattern, *n* (%)	1 (4.7)	-	0 (0.0)	-
UIP pattern, *n* (%)	3 (14.3)	-	21 (100.0)	-
Probable UIP pattern, *n* (%)	3 (14.3)	-	0 (0.0)	-
**Other SSc clinical manifestations**				
Renal impairment, *n* (%)	1 (4.8)	1 (5.0)	-	-
Cardiac involvement, *n* (%)	6 (28.6)	1 (5.0)	-	-
Raynaud’s phenomenon, *n* (%)	21 (100.0)	20 (100.0)	-	-
Esophageal dysfunction, *n* (%)	12 (57.1)	5 (25.0)	-	-
Calcinosis, *n* (%)	0 (0.0)	6 (30.0)	-	-
Synovitis, *n* (%)	6 (28.6)	6 (30.0)	-	-

SSc: systemic sclerosis; ILD: interstitial lung disease; IPF: idiopathic pulmonary fibrosis; SD: standard deviation; ANA: anti-nuclear antibodies; ACA: anti-centromere antibodies; ATA: anti-topoisomerase I antibodies; CRP: C-reactive protein; ESR: erythrocyte sedimentation rate; FVC: forced vital capacity; FEV1: forced expiratory volume in one second; DLCO: diffusing capacity of the lung for carbon monoxide; HRCT: high resolution computed tomography; UIP: usual interstitial pneumonia; NSIP: non-specific interstitial pneumonia.

**Table 2:** Correlation of EPC frequency with continuous variables related to demographic and disease features.

	SSc-ILD^+^ Patients		SSc-ILD^−^ Patients		IPF Patients	
	r	*p*	r	*p*	r	*p*
Age (years)	−0.33	0.14	−0.34	0.14	0.02	0.94
Duration of SSc disease (years)	**−0.45**	**0.04**	−0.10	0.69	-	-
CRP (mg/dL)	−0.08	0.76	−0.26	0.26	-	-
ESR (mm/1st hour)	−0.25	0.33	−0.01	0.96	-	-
FVC (% predicted)	−0.24	0.30	0.06	0.82	−0.21	0.35
FEV1 (% predicted)	−0.19	0.40	0.04	0.89	−0.19	0.40
FEV1/FVC (% predicted)	0.07	0.74	0.13	0.61	−0.05	0.83
DLCO (% predicted)	−0.01	0.99	−0.06	0.84	−0.23	0.40

EPC: endothelial progenitor cells; SSc: systemic sclerosis; ILD: interstitial lung disease; IPF: idiopathic pulmonary fibrosis; CRP: C-reactive protein; ESR: erythrocyte sedimentation rate; FVC: forced vital capacity; FEV1: forced expiratory volume in one second; DLCO: diffusing capacity of the lung for carbon monoxide. Significant results are highlighted in **bold**.

**Table 3:** Differences in EPC frequency according to categorical variables related to demographic and disease features.

Variable	Category	SSc-ILD^+^ Patients		SSc-ILD^−^ Patients		IPF Patients	
		Mean ± SD	*p*	Mean ± SD	*p*	Mean ± SD	*p*
Sex	Male	**0.045 ± 0.021**	**0.04**	0.047 ± 0.017	0.28	0.093 ± 0.047	0.70
	Female	**0.030 ± 0.007**		0.024 ± 0.028		0.086 ± 0.042	
Smoking ever	No	0.022 ± 0.018	0.50	0.020 ± 0.019	0.40	0.092 ± 0.048	0.93
	Yes	0.040 ± 0.014		0.031 ± 0.033		0.091 ± 0.045	
ANA	No	0.025	0.46	0.022 ± 0.017	0.85	-	
	Yes	0.037 ± 0.016		0.027 ± 0.029			
ACA	No	0.037 ± 0.016	0.33	0.024 ± 0.021	0.76	-	
	Yes	0.021		0.028 ± 0.036			
ATA (anti-Scl70)	No	0.033 ± 0.011	0.35	0.026 ± 0.030	0.93	-	
	Yes	0.040 ± 0.193		0.025 ± 0.023			
PH	No	0.033 ± 0.013	0.07	0.026 ± 0.028	-	0.088 ± 0.037	0.52
	Yes	0.052 ± 0.028		-		0.106 ± 0.066	
HRCT pattern	NSIP	0.032 ± 0.010	0.36	-		-	-
	UIP	0.039 ± 0.023				0.091 ± 0.045	

EPC: endothelial progenitor cells; SSc: systemic sclerosis; ILD: interstitial lung disease; IPF: idiopathic pulmonary fibrosis; SD: standard deviation; ANA: anti-nuclear antibodies; ACA: anti-centromere antibodies; ATA: anti topoisomerase I antibodies; PH: pulmonary hypertension; HRCT: high resolution computed tomography; NSIP: non-specific interstitial pneumonia; UIP: usual interstitial pneumonia. Significant results are highlighted in **bold**.

## Data Availability

All data generated or analyzed during this study are included in this published article.

## Supplementary Materials

The following is available online at https://www.mdpi.com/article/10.3390/biomedicines9070847/s1, Table S1: Differences in EPC frequency between SSc-ILD^+^ patients and the three comparative groups (healthy controls, SSc-ILD^−^ and IPF patients).
