# Supplementary material for: Endothelial Progenitor Cells: Relevant Players in the Vasculopathy and Lung Fibrosis Associated with the Presence of Interstitial Lung Disease in Systemic Sclerosis Patients
Source: Biomedicines. 2021 Jul 20;9(7):847. doi: 10.3390/biomedicines9070847 (PMC8301775; doi:10.3390/biomedicines9070847)
Supplement: Supplementary file 1 [file biomedicines-09-00847-s001.zip › biomedicines-1293587-supplementary.pdf]

biomedicines

Supplementary Materials

Endothelial Progenitor Cells: Relevant Players in the Vasculopathy and Lung Fibrosis Associated with the Presence of Interstitial Lung Disease in Systemic Sclerosis Patients

Verónica Pulito-Cueto[1*], Sara Remuzgo-Martínez[1*], Fernanda Genre[1*], Belén Atienza-Mateo[1,2,3], Víctor M. Mora-Cuesta[1,4], David Iturbe-Fernández[1,4], Leticia Lera-Gómez[1], Raquel Pérez-Fernández[1], Diana Prieto-Peña[1,3], Virginia Portilla[1,3], Ricardo Blanco[1,3], Alfonso Corrales[1,3], Oreste Gualillo[5], José M. Cifrián[1,4,6], Raquel López-Mejías[1] and Miguel A. González-Gay[1,3,6,7**]**

Table S1. Differences of EPC frequency between SSc-ILD$^+$ patients and the three comparative groups (healthy controls, SSc-ILD$^-$ and IPF patients).

	EPC frequency (Mean ± SD)	*p*
SSc-ILD$^+$ patients vs healthy controls	0.033 ± 0.012 vs 0.013 ± 0.010	**< 0.001**
SSc-ILD$^+$ patients vs SSc-ILD$^-$ patients	0.033 ± 0.012 vs 0.021 ± 0.017	**0.012**
SSc-ILD$^+$ patients vs IPF patients	0.033 ± 0.012 vs 0.087 ± 0.041	**< 0.001**

EPC: endothelial progenitor cells; SSc: systemic sclerosis; ILD: interstitial lung disease; IPF: idiopathic pulmonary fibrosis; SD: standard deviation. Significant results are highlighted in **bold**.
